# New Insights into TRP Ion Channels in Stem Cells

**DOI:** 10.3390/ijms23147766

**Published:** 2022-07-14

**Authors:** Jing Guo, Chang Shan, Jiao Xu, Mei Li, Jiayu Zhao, Wei Cheng

**Affiliations:** Institute of Cancer Stem Cell, Dalian Medical University, Dalian 116044, China; gjjing1020@163.com (J.G.); sc447066947@gmail.com (C.S.); xujiao0203@gmail.com (J.X.); limei791@dmu.edu.cn (M.L.); evezhao1108@gmail.com (J.Z.)

**Keywords:** TRPs, stemness, self-renewal, proliferation, cell cycle, calcium influx

## Abstract

Transient receptor potential (TRP) ion channels are cationic permeable proteins located on the plasma membrane. TRPs are cellular sensors for perceiving diverse physical and/or chemical stimuli; thus, serving various critical physiological functions, including chemo-sensation, hearing, homeostasis, mechano-sensation, pain, taste, thermoregulation, vision, and even carcinogenesis. Dysregulated TRPs are found to be linked to many human hereditary diseases. Recent studies indicate that TRP ion channels are not only involved in sensory functions but are also implicated in regulating the biological characteristics of stem cells. In the present review, we summarize the expressions and functions of TRP ion channels in stem cells, including cancer stem cells. It offers an overview of the current understanding of TRP ion channels in stem cells.

## 1. Introduction

Stem cells possess the capacity to self-renew; they show multilineage differentiation potential and can develop into more mature, specialized cells [1,2]. Stem cells can be classified as embryonic or adult stem cells, depending on their origins. Adult stem cells, such as hematopoietic stem cells, mesenchymal stem/stromal cells, endothelial progenitor cells, neural stem cells, colon stem cells, lung stem cells, epidermal stem cells, hair follicle bulge stem cells, etc., are present in all types of organs and tissues in diverse organisms [3]. Cancer stem cells (CSC) have been found in tumors and leukemia. CSCs are tumorigenic and are capable of producing all types of cells found in tumor tissues [4]. They are responsible for tumor maintenance, tumor metastasis, and tumor recurrence [5,6,7,8]. While the significance of stem cells in development, injury repair, and even carcinogenesis, is well-known, how stem cells respond to internal and environmental cues is not fully understood.

Transient receptor potential (TRP) channels are non-selective cation ion channels particularly noticeable for their physiological roles as cellular sensors. TRP channels on the plasma membrane are responsible for receiving signaling from other cells and the ambient environment and, hence, they play critical roles in stem cells. TRP ion channels are presumed tetramers formed of either identical or different subunits. Each TRP channel subunit has six transmembrane segments, with both N-terminal and C-terminal located in the intracellular compartment [9,10]. The mammalian TRP channel family consists of 28 members distributed in six subfamilies, including TRPC (canonical), TRPV (vanilloid), TRPM (melastatin), TRPML (mucolipin), TRPA (ankyrin), and TRPP (polycystin). Intracellular calcium signaling is crucial for modulating stem cell functions. Most TRP channels are Ca^2+^ permeable, which can directly trigger Ca^2+^ influx. Thus, they may be involved in regulating stem cell functions.

TRP ion channels are mainly expressed in neuronal and non-neuronal cells and tissues. As molecular sensors, TRP ion channels exhibit diverse activation mechanisms with physiological and pathophysiological implications [11,12]. TRP ion channels respond to changes in the external environment; they are also involved in regulating the cellular microenvironment. Given the functional properties of TRP channels and their expressions in stem cells, it is not surprising that they may participate in regulating stem cell physiology and differentiation. Indeed, recent studies have found that TRP ion channels are implicated in stem cells [13,14,15]. This review summarizes the expression patterns of TRP channels in various stem cells, with particular focus on the potential roles TRP channels play in regulating stem cell physiology and pathophysiology.

## 2. TRPs in Stem Cells

The *Trp* gene was first cloned from Drosophila in 1989; in the following years, mammalian TRP channels were identified and characterized. The presence of the TRPC complex in the central nervous system (CNS) development sparked the research of TRPs in stem cells [16,17]. To date, TRP channels (TRPCs, TRPMs, TRPVs, TRPA, TRPMLs) are distributed in stem cells, including mesenchymal stem cells (MSC), neural stem cells (NSC), dental pulp stem cells (DPSC), trophoblast stem cells (TSC), embryonic stem cells (ESC), intestinal stem cells (ISC), etc. (Table 1).

### 2.1. TRPCs

The TRPC subfamily comprises seven members—TRPC1, TRPC2, TRPC3, TRPC4, TRPC5, TRPC6, and TRPC7.

TRPC1 has been abundantly expressed in C2C12 mouse skeletal myoblasts and upregulated in the differentiation of myoblasts [18,19]. It suggests a crucial role for TRPC1 in myogenesis.

In a study by Fiorio Pla et al., TRPC1, 2, 3, 4, and 6 transcripts were broadly expressed in embryonic rat NSCs. Furthermore, TRPC1 co-expressed with fibroblast growth factor receptor (FGFR)-1 in proliferating NSC-derived progeny in the presence of the basic fibroblast growth factor (bFGF) [16]. In addition, the store-operated channel complexes of TRPC1, Orai1, and STIM1 were co-expressed in mouse NSCs [20].

TRPC1, 2, 3, 4, 5, and 6 mRNAs were observed in MSCs derived from human tissues or the bone marrow of rabbits [21,22,23]. Only the TRPC1 protein was detected by both immunofluorescence staining and western blot analysis in these cells [23].

Researchers found TRPC1 and TRPC6 expressed in rat bone marrow stromal cells (BMSCs) [24].

TRPC1, 3, 6, and 7 transcripts were detected in CD34^+^ stem cells isolated from human umbilical cord blood. During stem cell differentiation to megakaryocytes, TRPC6 expression was found significantly increased [25,26].

### 2.2. TRPMs

The TRPM channel subfamily includes eight members (TRPM1, TRPM2, TRPM3, TRPM4, TRPM5, TRPM6, TRPM7, and TRPM8).

TRPM2 has been identified in fetal mouse NSCs [27].

Dental pulp harbors subpopulations of stem cells that are capable of differentiating via several pathways, fulfilling a function in tissue damage. TRPM4 channels are permeable to monovalent cations, and their presence in rat DPSCs introduces voltage dependency and Na^+^ conductivity to these cells [28]. Moreover, it has been revealed that voltage-gated TRPM7 is widely expressed in the cell membranes and cytoplasms of human DPSCs [29].

TRPM7 channels are abundantly expressed in mouse TSCs as well as mouse ESCs; in comparison, TRPM4 shows a lower distribution in mouse TSCs [30].

Among mammalian TRP ion channels, both TRPM6 and TRPM7 have a C-terminal kinase, which is combined with Mg^2+^ and Mg^2+^-ATP to attenuate the activities of TRPM7 with Mg^2+^ or Ca^2+^ permeation [31]. TRPM7 has been found present in both mouse and human bone marrow-derived MSCs, and TRPM7 mRNA (but not TRPM6) is upregulated during osteogenesis [32,33]. It should be noted that TRPM7 is involved in mediating Mg^2+^ permeation in mouse MSCs, yet its mechanical stimulation involves facilitated Ca^2+^ permeation in human MSCs [34,35,36].

Besides internal Mg^2+^ and Ca^2+^, cytosolic pH (protons) also mediates the TRPM7 channel activity in mouse BMSCs [37].

### 2.3. TRPVs

The TRPV subfamily is composed of six members (TRPV1, TRPV2, TRPV3, TRPV4, TRPV5, and TRPV6).

TRPV1 plays a prominent role in sensory neurons, exhibiting functions in inflammation and nociception in the peripheral nervous system and somewhat ambiguous implications in the central nervous system [38,39]. Ramírez-Barrantes et al. detected signals of immune-fluorescent TRPV1 in rat NSCs [40]. Further, TRPV1 has been found expressed in human NSCs and upregulated in differentiated NSCs [41]. In neural crest-like stem cells from a neonatal mouse epidermis, TRPV1 has been detected and is shown to be activated by capsaicin [42]. Moreover, TRPV1 could traffic rapidly from intracellular localizations to the plasma membrane upon stimulation of the nerve growth factor (NGF) in dorsal root ganglia [43]. On the other hand, the vascular endothelial growth factor (VEGF) can downregulate the expression of TRPV1 in rat NSCs [44].

The expression of TRPV1 has been identified during human DPSC differentiation toward neurons, where activation of this channel is suggested to play an important role [45].

In addition, recent studies have identified the presence of TRPV1 in human adipose-derived stem cells and myogenic cells [46,47].

In mouse TSCs, a strong expression of TRPV2 has been observed, while TRPV4 exhibits a lower expression. The expression of TRPV2 increases during TSC differentiation [30].

TRPV4 is permeable to monovalent and divalent cations, but with higher affinities to Ca^2+^ and Mg^2+^ than Na^+^ cations [48,49]. Studies have found that TRPV4 channels are expressed in the murine mesenchymal stem cell line C3H10T1/2 as well as human and mouse MSCs isolated from bone marrow [50,51,52].

Jin et al. uncovered TRPV4 expression in periodontal stem cells and demonstrated that activation of TRPV4 by mechanical force could induce cell proliferation, with accumulation of inflammatory cytokines of IL-1β, TNF-α, IL-6 and MCP-1 [53].

### 2.4. TRPA1

The TRPA subfamily only contains one member (TRPA1).

TRPA1 is remarkably localized in the ISCs of drosophila [54].

Recently, it has been found that TRPA1 is expressed in human DPSCs, and intracellular ROS could upregulate TRPA1 distribution in vitro and in vivo [55].

TRPA1 is also expressed in human MSCs and NSCs, and is upregulated in differentiated NSCs [21,41].

### 2.5. TRPMLs

The TRPML subfamily consists of three members—TRPML1 TRPML2, and TRPML3.

The transcripts of TRPML1 and TRPML2 have been detected in MSCs and NSCs, but their roles in these cells are lacking [15,21].

**Table 1:** TRPs distributed in stem cells.

TRPs	Stem Cells	Origins	References
TRPC1	MSCs	Rabbit bone marrow	[23]
	MSCs	Human tissue and cells	[21,22]
	BMSCs	Rat bone marrow	[24]
	NSCs	Rat embryos	[16]
	NSCs	Mouse	[20]
	CD34+ stem cells	Human cord blood	[25]
	C2C12 myoblasts	Mouse skeleton	[18,19]
TRPC2	MSCs	Rabbit bone marrow	[23]
	NSCs	Rat embryos	[16]
TRPC3	MSCs	Human tissues	[21]
	NSCs	Rat embryos	[16]
	CD34+ stem cells	Human cord blood	[25]
TRPC4	MSCs	Human tissues	[21]
	MSCs	Rabbit bone marrow	[23]
	NSCs	Rat embryos	[16]
TRPC5	MSCs	Human tissues	[21]
TRPC6	MSCs	Human tissues	[21]
	MSCs	Rabbit bone marrow	[24]
	NSCs	Rat embryos	[16]
TRPC7	CD34^+^ stem cells	Human cord blood	[25]
TRPM2	NSCs	Mouse embryos	[27]
TRPM4	DPSCs	Rat	[28]
	TSCs	Mouse	[30]
TRPM7	DPSCs	Human	[29]
	TSCs	Mouse	[30]
	ESCs	Mouse	[30]
	MSCs	Mouse bone marrow	[33]
	MSCs	Human bone marrow	[32,34,35,36]
	BMSCs	Mouse	[37]
TRPV1	NSCs	Rat	[40,44]
	NSCs	Human	[41]
	Neural crest-like stem cells	Mouse	[42]
	DPSCs	Human	[45]
	Adipose-derived stem cells	Human	[47]
	Myogenic cells	Mouse	[46]
TRPV2	TSCs	Mouse	[30]
TRPV4	TSCs	Mouse	[30]
	MSCs	Mouse	[50,52]
	MSCs	Human	[51]
	Periodontal stem cells	Rat	[53]
TRPA1	ISCs	Drosophila	[54]
	DPSCs	Human	[55]
	MSCs	Human	[21]
	NSCs	Human	[41]
TRPML1	MSCs	Human tissues	[21]
TRPML2	NSCs	Human	[15]

## 3. TRPs in Cancer Stem Cells (CSCs)

CSCs are villains in cancer origination and development. They account for most cancer malignancies and recurrences. So far, members of TRPC, TRPM, TRPV, and TRPA subfamilies have been characterized in CSCs (Table 2).

### 3.1. TRPCs

Early evidence showed that TRPC1 presented in glioblastoma stem cells (GSC) with store-operated channel proteins of Orai1 and STIM1 [56].

### 3.2. TRPMs

TRPM7 has been found in GSCs derived from the human glioblastoma cell line A172 [57].

Moreover, increasing TRPM7 mRNA has been observed in tumor spheres derived from human lung cancer cells, accompanied by enhanced expression of the cancer stem cell markers SOX2, CD133, and KLF4 [58].

### 3.3. TRPVs

TRPV1 has been observed in GSCs and is upregulated in differentiated GSCs of proneural-like and mesenchymal-like cells in an ERK-dependent manner [41]. Nabissi et al. have demonstrated that glioblastoma and GSCs selectively express the TRPV1 5′ untranslated region variant 3 (TRPV1V3), where one of four variants is produced by selective splicing of the first exon. In addition, increased expression of TRPV1V3 was found by inducing differentiation of GSCs [59], suggesting that the TRPV1 variant 3 can be a potential prognostic marker for glioma.

TRPV2 is present in glioma and GSCs isolated from adult patients. Differentiation of GSCs facilitates the incremental accumulation of TRPV2 expression [60,61].

Hepatocellular stem cells are partially accountable for the highest mortality rate in liver cancer. Hu et al. reported the elevated expression of TRPV2 in established hepatocellular carcinoma cell lines. There is a positive correlation between stemness and TRPV2 expression levels [62].

### 3.4. TRPA1

Research indicates that TRPA1 is expressed in human GSCs and its expression increases during the differentiation of GSCs [41].

TRPA1 has been observed to be overexpressed in the MSCs of human lung cancer tissues, which might be related to the poor prognosis of non-small cell lung cancer [63]. The finding indicates that TRPA1 may serve as a potential prognosis marker and facilitate the search for the target drug to inhibit the progression of non-small cell lung cancer.

**Table 2:** TRPs expressed in cancer stem cells.

TRPs	Cancer Stem Cells	Origins	References
TRPC1	GSCs	Human tumor tissues	[56]
TRPM7	GSCs	Human glioblastoma cells	[57]
	Tumor spheres	Human lung cancer cells	[58]
TRPV1	GSCs	Human tumor tissues	[41]
TRPV2	GSCs	Human Adult patients	[60,61]
	Stem-like cells	Human liver cancer cells	[62]
TRPA1	GSCs	Human	[41]
	MSCs	Human lung cancer tissues	[63]

## 4. TRPs in Progenitor Cells

TRP channels are mainly cell sensors prominently distributed in the nervous system. In neural progenitor cells (NPC), almost all TRP channel subfamilies have been detected. Moreover, TRPs distribute in endothelia progenitors, hematopoietic progenitors, myeloid precursors, and osteoclast precursors (Table 3).

### 4.1. TRPCs

NPCs are precursor cells presenting in the process of differentiation of NSCs toward neurons. To date, TRP ion channels are observed in NPCs with relatively high permeability to calcium. Studies have found that mRNAs for TRPC1, 3, 4, 5, and 6 are present in both A2B5^+^ (neural cell surface antigen) NPCs and differentiated neuronal cells. In A2B5^+^ NPCs isolated from SD rats, there were upregulations of TRPC1 and TRPC4 gene expressions compared to the differentiated cells, while TRPC3, TRPC5, and TRPC6 a downregulated compared to differentiated cells [64]. Louhivuori et al. performed research on embryonic mouse NPCs and found that corresponding to the motor patterns of NPCs, TRPC1 and TRPC3 genes were expressed during cell proliferation [65]. Another study elucidated that TRPC1 was detected, along with Orai1 and STIM1, in NPCs isolated from the hippocampal tissue of an adult male mouse [66]. In human NPCs derived from fetal midbrain tissue, TRPC1, 3, 4, 5, and 6 were found expressed during NPC proliferation and differentiation [67].

As the precursors of vascular endothelial cells, endothelial progenitor cells (EPC) could be recruited to repair blood vessels in vascular injury. With the distribution of TRPC1 on the plasma membranes of EPCs, this channel appears to be involved in regulating cell migration and angiogenesis via the signaling pathway of Calmodulin/eNOS [68,69].

Ong and co-workers found TRPC1 expressed in hematopoietic progenitors, myeloid precursors, and osteoclasts precursors, and regulated osteoclastogenesis via store-operated Ca^2+^ entry [70].

### 4.2. TRPVs

Notably, TRPV1, TRPV2, and TRPV3 trigger Ca^2+^ influx in human NPCs. During cell differentiation, the expressions of TRPV2 and TRPV3 have been remarkably downregulated [71].

Further, NSCs and NPCs induced by somatic mutations are considered to be the sources of high-grade astrocytoma. Stock et al. demonstrated that TRPV1 was overexpressed in high-grade astrocytomas, and activation of TRPV1 could induce tumor cell death. NPCs in proximity are the main sources of releasing the endogenous TRPV1 agonist [72], so TRPV1 may be a potential target for therapy in high-grade astrocytomas.

In addition, TRPV4, 5, and 6 are functionally expressed in human NPCs [67].

Moreover, TRPV4 has been observed in EPCs [73].

### 4.3. TRPMs and Other TRPs

TRPM2, 3, 4, and 7 have been identified as distributed in mouse and human NPCs; in particular, activation of TRPM3 could induce calcium influx [27,67].

In addition, TRPML1, TRPML2, and TRPP2 are expressed in human NPCs derived from fetal midbrain tissues [15,67].

**Table 3:** TRPs distributed in progenitor cells.

TRPs	Progenitor Cells	Origins	References
TRPC1	Hematopoietic progenitors	Mouse bone marrow	[70]
	Myeloid precursors	Mouse bone marrow	[70]
	Osteoclasts precursors	Mouse bone marrow	[70]
	NPCs	Rat	[64]
	NPCs	Mouse	[65]
	NPCs	Human	[67]
	EPCs	Rat	[68]
	EPCs	Mouse	[69]
TRPC3	NPCs	Rat	[64]
	NPCs	Mouse	[65]
	NPCs	Human	[67]
TRPC4	NPCs	Rat	[64]
	NPCs	Human	[67]
TRPC5	NPCs	Rat	[64]
	NPCs	Human	[67]
TRPC6	NPCs	Rat	[64]
	NPCs	Human	[67]
TRPV1	NPCs	Human	[71]
TRPV2	NPCs	Human	[71]
TRPV3	NPCs	Human	[71]
TRPV4	NPCs	Human	[67]
	EPCs	Human	[73]
TRPV5	NPCs	Human	[67]
TRPV6	NPCs	Human	[67]
TRPM2	NPCs	Human	[67]
	NPCs	Mouse	[27]
TRPM3	NPCs	Human	[67]
TRPM4	NPCs	Human	[67]
TRPM7	NPCs	Human	[67]
TRPML1	NPCs	Human	[67]
TRPML2	NPCs	Human	[15]
TRPP2	NPCs	Human	[67]

## 5. TRP Roles in Stem Cells

TRP channels mediate Ca^2+^ entry from extracellular compartments or they release from internal stores to stimulate numerous Ca^2+^ signaling and control diverse cellular processes. Based on their distributions in stem cells and cancer stem cells, TRPs are involved in modulating cell cycles, cell proliferation and differentiation, cell migration, and self-renewal of these cells.

### 5.1. TRPCs

TRPC channels are mainly characterized as receptor-operated channels that can be activated by either intracellular messengers or by depletion of internal Ca^2+^ stores. Studies have identified that TRPCs can interact with STIM1 and Orai1 components to constitute store-operated Ca^2+^ entry. Regarding the complex of TRPC1 with STIM1 and Orai1 in NSCs, NPCs and GSCs may contribute to store-dependent Ca^2+^ signaling [20,56,66].

In rat BMSCs, the resting membrane potential shows cycle-dependent changes, with the highest depolarization in the G1 phase and hyperpolarization in the S phase. Such cycle-dependent changes of resting membrane potential are fully matched with the upregulation of TRPC6 in the G1 phase and downregulation of TRPC6 in the S phase. Knockdown of TRPC6 reduces the resting membrane potential, which promotes the progression of the cell cycle. Therefore, TRPC6 may act as store-operated channels to regulate the cell cycles of BMSCs by changing the membrane potential via modulating the Ca^2+^ entry [24].

In NSCs, Ca^2+^ signaling modified by TRPCs promotes cell proliferation and differentiation. Specifically, TRPC1 potentiates bFGF/FGFR-1-induced Ca^2+^ influx in rat embryonic NSCs, which in turn promotes the proliferation of embryonic NSCs [16]. In addition, the thyroid hormone can induce significant Ca^2+^ influx through TRPC1 in ventral midbrain NSCs, thereby regulating the differentiation of NSCs [74]. The expression of TRPC5 increases with elevated store-operated Ca^2+^ entry during the differentiation of A2B5^+^ NPCs, promoting neuronal differentiation [64].

**Figure 1:**
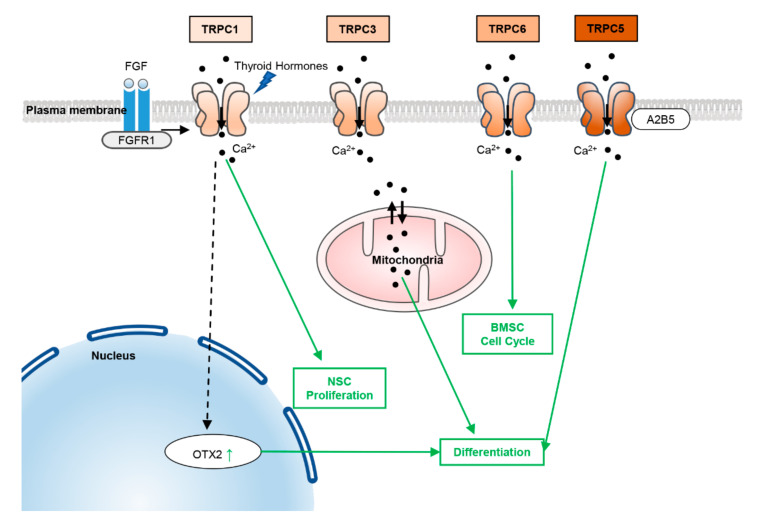
TRPCs function in stem cells. TRPC1-induced Ca^2+^ influx activates the FGF/FGFR1 signaling pathway or OTX2 to promote proliferation or differentiation of NSCs. TRPC3 triggers Ca^2+^ influx in mitochondria, which promotes the stabilization of differentiation processes of ESCs to NSCs. High expression of TRPC5 in A2B5^+^ NPCs with increased store-operated Ca^2+^ entry promotes cell differentiation. Further, the dynamic distribution of TRPC6 can control the magnitude of store-operated Ca^2+^ entry via changes in membrane potential to regulate the cell cycle of BMSCs.

Insights from Hao et al. found that TRPC3 is expressed in mouse ESCs. Activation of TRPC3 could promote the Ca^2+^ influx in mitochondria, maintain the integrity of mitochondrial membrane proteins of ESCs and, therefore, stabilize the differentiation process of ESCs to NSCs. Knockout of TRPC3 destroys mitochondrial membrane proteins and induces apoptosis in undifferentiated ESCs [75] (Figure 1).

### 5.2. TRPMs

TRPM channels encode nonselective cation channels involved in many biological roles, including thermosensation, taste, cell migration, Mg^2+^ homeostasis, and reabsorption. So far, TRPMs are found distributed in MSCs, NSCs, NPCs, DPSCs, GSCs, and lung cancer stem-like cells and, hence, are implicated in these cells.

Studies indicate that TRPMs mediated stem cell differentiation and other biological roles via different signaling pathways with Ca^2+^ involvement. The Ca^2+^ influx induced by TRPMs can activate different cellular processes or adjust gene transcription. 

Cheng et al. found that TRPM7 upregulated in differentiated MSCs [33]. During this differentiation process, the Ca^2+^ influx mediated via TRPM7 induces the activation of the phospholipase C (PLC) signaling pathway, promotes Ca^2+^ release into the cytoplasm from the endoplasmic reticulum (ER), then the nuclear factor of activated T cells c1 (NFATc1) translocates to the nucleus, promoting osteogenesis in human bone marrow MSCs [36]. Moreover, TRPM7 regulates SMAD1 activity by phosphorylating PLC [32]. Activation of PLC leads to recruitment of PKC and calmodulin (CaM) with stimulation of CaM kinase II, which then phosphorylates SMAD1 and induces its translocation to the nucleus, thereby activating osteogenic genes Runx2, Osterix, and OCN to promote osteogenesis [76]. 

In human bone marrow MSCs, TRPM8 shows specific expression in the ER, suggesting that its main function may be related to the release of intracellular Ca^2+^. In addition, activation of TRPM8 promotes osteogenic differentiation of human MSCs [14]. In rat dental follicle stem cells, functional expression of TRPM4 facilitates adipogenesis via Ca^2+^ signaling [77]. The expression of TRPM2 channels is increased during heat stress in NSCs. In hyperthermia, knockout of TRPM2 reduces NPC proliferation and accelerates premature neuronal differentiation. TRPM2 regulates the self-renewal of NPCs by targeting the transcription factor of specificity protein 5 (SP5). Briefly, increased Ca^2+^ influx through TRPM2 by heat stimuli inhibits the phosphorylation of β-catenin, accumulating β-catenin, then binds to the SP5 promoter and activates the gene transcription process to ultimately promote NPC proliferation [27]. In contrast, activation of TRPM3 and TRPV4 via agonism shows no effects on the proliferation and survival of cultured human NPCs [67].

In CSCs, TRPM7 activates STAT3, which in turn binds to acetaldehyde dehydrogenase (ALDH)-1 promoter to upregulate the expression of ALDH1, a marker of GSCs, thus facilitating the self-renewal and differentiation of GSCs. Furthermore, TRPM7 activates Notch signaling pathways and promotes the expression of CD133 and ALDH1, enhancing glioma stemness [57,78]. Similarly, upregulated TRPM7 enhances the lung cancer stem cell-like phenotype and promotes lung cancer metastasis via the Hsp90α/uPA/MMP2 signaling pathway [58]. TRPM7 also maintains the stem cell features of neuroblastoma by regulating the expression of the Snail family transcriptional repressor 2 (SNAI2) transcription factor, which is involved in epithelial–mesenchymal transitions and has antiapoptotic activity [79,80]. Further studies need to determine the molecular mechanisms of TRPM7-driven SNAI2 to control stem cell features.

**Figure 2:**
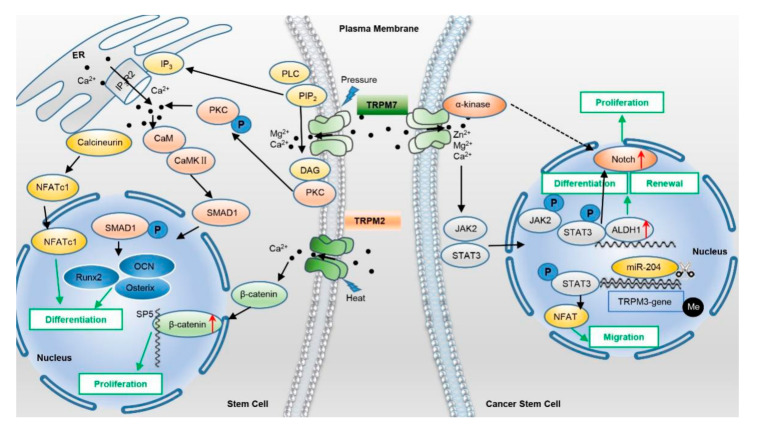
TRPMs function in stem cells and cancer stem cells via different signaling pathways. In MSCs, the Ca^2+^ influx via TRPM7 triggers the activation of the PLC signaling pathway, subsequently releases Ca^2+^ from ER, and induces nuclear translocation of NFATc1, promoting differentiation in human bone marrow MSCs. Moreover, activation of PLC leads to phosphorylation of PKC and its intracellular translocation, recruitment of CaM, and stimulation of CaMKII, which then phosphorylates SMAD1 and induces its translocation to the nucleus, thereby activating osteogenic genes Runx2, Osterix, and OCN to promote MSC differentiation and osteogenesis. Activation of TRPM2 by heat stress elevates intracellular calcium influx, which inhibits the phosphorylation of β-catenin and results in the augment of β-catenin on the SP5 promoter, promoting NPC proliferation. In GSCs, activation of TRPM7 activates the JAK2-STAT3 signaling pathway, which phosphorylates JAK2 and STAT3 and induces the translocation of JAK2 and STAT3 to the nucleus; STAT3 then activates ALDH1 via binding to its promoter and/or activates Notch signaling, enhancing GSC differentiation, renewal, and proliferation. Downregulation of miR-204 via methylation of the promoter of its host gene TRPM3 could activate the STAT3-NFAT pathway, promoting GSC invasion and stem cell-like phenotype.

Mg^2+^ and microRNA have been found implicated in TRP modulation in stem cell functions. Specifically, TRPM7 regulates cell proliferation in human DPSCs via intracellular Mg^2+^ signaling. In the absence of TRPM7, cell proliferation is significantly subdued; as a result, the cells are mainly arrested in the G0/G1 phase. Moreover, inhibition of TRPM7 suppresses cell migration, and downregulation of TRPM7 in human DPSCs inhibits osteogenic differentiation. These suggest that TRPM7 may play a role in the dental pulp repair process [29]. MiR-204 is an intron miRNA located between exons 7 and 8 of the *TRPM3* gene. The reduction of miR-204 due to the high methylation of its host gene *TRPM3* in gliomas can promote cell migration and enhance cell stemness [81]. It has been demonstrated that TRPM3 could interact with STAT3, with activation of STAT3 suppressing miR-204 expression. Furthermore, downregulation of miR-204 leads to the activation of the Src-STAT3-NFAT signaling pathway in pulmonary artery smooth muscle cells [82]. STAT3 also directly facilitates NFAT expression. Hence, the silence of miR-204 may promote GSC migration via the Ca^2+^-sensitive STAT3-NFAT pathway (Figure 2).

### 5.3. TRPVs

TRPVs are the main pathways of Ca^2+^ entry in stem cells and have been involved in mediating cell differentiation and proliferation [71]. TRPV1 can be activated by light in the blue (415 nm) and green (540 nm) wavelength range, inhibiting cell proliferation in adipose-derived stem cells [47]. TRPV2 is upregulated in the differentiation of mouse TSCs. It suggests that TRPV2 may be involved in placental development [30]. It has been found that far-infrared radiation inhibits the adipogenic differentiation of tonsil-derived MSCs by inducing intracellular Ca^2+^ mobilization [83]. Further studies have shown that the TRPV2 ion channel acts as a receptor of far-infrared radiation and mediates the inhibition process in the adipogenic differentiation of MSCs [84]. Hu et al. reported that flow shear stress activates TRPV4 in MSCs, follows Ca^2+^ influx, and induces early osteogenic differentiation [50,85]. In addition, TRPV4-mediated Ca^2+^ signaling is crucial for the formation of collagen in bone marrow MSCs [51]. Furthermore, Ca^2+^/calcineurin/NFAT signaling involves bone development and regeneration [86,87]. One study has revealed that TRPV1 deletion in BMSCs can downregulate the expression and nuclear translocation of NFATc1, and, thus, impair osteoclastogenesis and osteogenesis [88]. TRPV4, in vitro or in vivo, promotes the proliferation of mature endothelial cells by activating a series of calcium-dependent transcription factors, reshaping the vascular network of injured tissues [89,90].

In cancer stem cells, TRPV2 inhibits GSC proliferation both in vitro and in vivo. Activation of TRPV2 by cannabidiol could trigger GSC differentiation and activate the autophagic process. During GSC differentiation, the upregulated splicing variant of acute myeloid leukemia 1a (AML-1a) directly affects the expression of TRPV2 via binding to the gene promoter of *TRPV2*, establishing a positive feedback circuit to promote cell differentiation [60]. In adult glioma patients, TRPV2 expression increases during GSC differentiation. Indeed, silencing or inhibiting TRPV2 can affect the differentiation of GSCs [61].

Over-expression of TRPV2 can attenuate the stemness of hepatoma SMMC-7721 cells. In contrast, knockout of TRPV2 can significantly increase cancer stem cell markers (CD133, CD44, and ALDH1) and enhance spheroid and colony formation in human hepatoma HepG2 cells [62]. However, the precise signaling mechanisms remain largely elusive.

### 5.4. TRPA1

**Figure 3:**
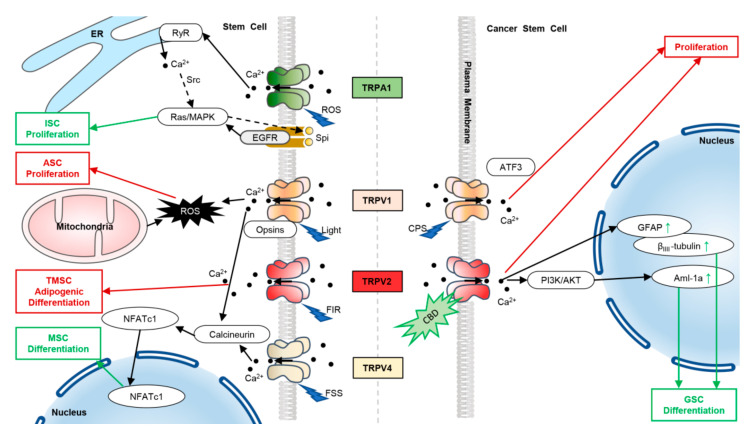
TRPVs and TRPA1 involved in modulating cell proliferation and differentiation in stem cells and cancer stem cells. In stem cells, activation of TRPA1 by ROS induces Ca^2+^ influx, which activates the RyR channel to release Ca^2+^ from ER to cytosol in ISC. The increased Ca^2+^ activates Ras/MAPK signaling via Src, and further amplifies Ras/MAPK signaling by autocrine Spi-EGFR signaling. The activated EGFR-Ras/MAPK signaling then induces the proliferation of ISCs. Blue (415 nm) and green (540 nm) wavelengths of light could activate TRPV1 and increase Ca^2+^ and ROS, thereby inhibiting cell proliferation in adipose-derived stem cells (ASC). Knockout of TRPV1 suppresses the expression and activities of NFATc1, leading to decreased osteoclast and osteoblast differentiation. Upon stimuli of far-infrared radiation (FIR), TRPV2 induces intracellular Ca^2+^ increase and inhibits tonsil-derived MSC (TMSC) differentiation. Flow shear stress (FSS) activates TRPV4 and induces Ca^2+^ influx, which mediates NFATc1 nuclear translocation, thereby promoting the osteogenic differentiation of MSCs. In cancer stem cells, TRPV1 with stimulation of capsaicin (CPS) inhibits the proliferation of NSCs and NPC-derived high-grade astrocytoma in an activating transcription factor 3 (ATF3)-dependent manner. Upon cannabidiol (CBD) application, AKT activity in GSCs has been inhibited by decreasing pAKT levels, leading to autophagy in GSCs. Meanwhile, the expressions of TRPV2 and Aml-1a, which bind to TRPV2 promoters, are both upregulated, and increased TRPV2 enriches the expression of glial fibrillary acidic protein (GFAP) and β_III_-tubulin, enhancing the differentiation of GSCs via the CBD-mediated autophagic process. Moreover, the activated TRPV2 inhibits GSC proliferation. Green arrows represent enhancement, red arrows represent inhibition.

TRPA1 is a well-known cytoplasmic Ca^2+^ regulator. It could upregulate cytosolic Ca^2+^ in ISCs to amplify and activate EGFR-Ras/MAPK signaling, which in turn drives ISC proliferation to maintain tissue homeostasis in the mid-gut of adult drosophila [54,91,92] (Figure 3).

## 6. Perspectives and Outlook

TRP ion channels in stem cells have been explored in almost five subfamilies but TRPPs. TRPPs contain three members (TRPP2, TRPP3, and TRPP5). TRPP2 has been demonstrated as an active coordinator in TRP channel heteromerization. Specifically, TRPP2 physically interacting with polycystin-1 (PKD1) has been identified [93]. A study reported that PKD1 was present in stem cells of variable origins. In addition, over-expression of PKD1 enhanced cell mobility and differentiation in umbilical cord blood-derived stem cells [94]. Wide-spread interaction and assembly in TRPs enhance the novel channel maturation and intracellular translocation [95]. It appears that many functional TRPs are distributed in stem cells. However, knowledge about the co-assembly of TRPs in stem cells is lacking. Further research regarding the heteromerization of TRPs in stem cells should be considered.

Although certain aspects of TRP ion channels in stem cells have been deciphered through independent studies in the past decade, the roles of TRP ion channels and their implications in stem cells need further investigation. With the application of single-cell sequencing and high-throughput screening, the expression patterns of TRP channels in stem cells will be better understood. In addition, the usage of reporter genes and animal models will provide the precise mechanisms of TRP channels involved in stem cells.

TRP channels are expressed/function in stem cells, including cancer stem cells, which may lead to new features of TRPs and stimulate further explorations, ranging from molecular biology to clinical research and even cosmetics. TRPs, as multimode cellular sensors, have been modulated by a wealth of natural compounds, which can be used as potential pharmacological targets for fundamental research and drug discovery.

## Data Availability

Not applicable.

## References

[1] Weissman I.L. (2000). Stem cells: Units of development, units of regeneration, and units in evolution. Cell.

[2] Chagastelles P.C., Nardi N.B. (2011). Biology of stem cells: An overview. Kidney Int. Suppl..

[3] Alison M.R., Islam S. (2009). Attributes of adult stem cells. J. Pathol..

[4] Yu J., Hu K., Smuga-Otto K., Tian S., Stewart R., Slukvin I.I., Thomson J.A. (2009). Human induced pluripotent stem cells free of vector and transgene sequences. Science.

[5] Kim C.F., Dirks P.B. (2008). Cancer and stem cell biology: How tightly intertwined?. Cell Stem Cell.

[6] Jordan C.T. (2004). Cancer stem cell biology: From leukemia to solid tumors. Curr. Opin. Cell Biol..

[7] Pardal R., Clarke M.F., Morrison S.J. (2003). Applying the principles of stem-cell biology to cancer. Nat. Rev. Cancer.

[8] Dick J.E. (2008). Stem cell concepts renew cancer research. Blood.

[9] Nilius B., Voets T. (2005). TRP channels: A TR(I)P through a world of multifunctional cation channels. Pflug. Arch..

[10] Clapham D.E. (2007). SnapShot: Mammalian TRP channels. Cell.

[11] Clapham D.E. (2003). TRP channels as cellular sensors. Nature.

[12] Zheng J. (2013). Molecular mechanism of TRP channels. Compr. Physiol..

[13] Chinigo G., Castel H., Chever O., Gkika D. (2021). TRP Channels in Brain Tumors. Front. Cell Dev. Biol..

[14] Henao J.C., Grismaldo A., Barreto A., Rodriguez-Pardo V.M., Mejia-Cruz C.C., Leal-Garcia E., Perez-Nunez R., Rojas P., Latorre R., Carvacho I. (2021). TRPM8 Channel Promotes the Osteogenic Differentiation in Human Bone Marrow Mesenchymal Stem Cells. Front. Cell Dev. Biol..

[15] Morelli M.B., Nabissi M., Amantini C., Tomassoni D., Rossi F., Cardinali C., Santoni M., Arcella A., Oliva M.A., Santoni A. (2016). Overexpression of transient receptor potential mucolipin-2 ion channels in gliomas: Role in tumor growth and progression. Oncotarget.

[16] Fiorio Pla A., Maric D., Brazer S.C., Giacobini P., Liu X., Chang Y.H., Ambudkar I.S., Barker J.L. (2005). Canonical transient receptor potential 1 plays a role in basic fibroblast growth factor (bFGF)/FGF receptor-1-induced Ca^2+^ entry and embryonic rat neural stem cell proliferation. J. Neurosci..

[17] Strubing C., Krapivinsky G., Krapivinsky L., Clapham D.E. (2003). Formation of novel TRPC channels by complex subunit interactions in embryonic brain. J. Biol. Chem..

[18] Formigli L., Sassoli C., Squecco R., Bini F., Martinesi M., Chellini F., Luciani G., Sbrana F., Zecchi-Orlandini S., Francini F. (2009). Regulation of transient receptor potential canonical channel 1 (TRPC1) by sphingosine 1-phosphate in C2C12 myoblasts and its relevance for a role of mechanotransduction in skeletal muscle differentiation. J. Cell Sci..

[19] Louis M., Zanou N., Van Schoor M., Gailly P. (2008). TRPC1 regulates skeletal myoblast migration and differentiation. J. Cell Sci..

[20] Domenichini F., Terrie E., Arnault P., Harnois T., Magaud C., Bois P., Constantin B., Coronas V. (2018). Store-Operated Calcium Entries Control Neural Stem Cell Self-Renewal in the Adult Brain Subventricular Zone. Stem Cells.

[21] Goralczyk A., van Vijven M., Koch M., Badowski C., Yassin M.S., Toh S.A., Shabbir A., Franco-Obregon A., Raghunath M. (2017). TRP channels in brown and white adipogenesis from human progenitors: New therapeutic targets and the caveats associated with the common antibiotic, streptomycin. FASEB J..

[22] Parate D., Franco-Obregon A., Frohlich J., Beyer C., Abbas A.A., Kamarul T., Hui J.H.P., Yang Z. (2017). Enhancement of mesenchymal stem cell chondrogenesis with short-term low intensity pulsed electromagnetic fields. Sci. Rep..

[23] Torossian F., Bisson A., Vannier J.P., Boyer O., Lamacz M. (2010). TRPC expression in mesenchymal stem cells. Cell. Mol. Biol. Lett..

[24] Ichikawa J., Inoue R. (2014). TRPC6 regulates cell cycle progression by modulating membrane potential in bone marrow stromal cells. Br. J. Pharm..

[25] Ramanathan G., Mannhalter C. (2016). Increased expression of transient receptor potential canonical 6 (TRPC6) in differentiating human megakaryocytes. Cell Biol. Int..

[26] Raghuwanshi S., Dahariya S., Sharma D.S., Kovuru N., Sahu I., Gutti R.K. (2020). RUNX1 and TGF-beta signaling cross talk regulates Ca(^2+^) ion channels expression and activity during megakaryocyte development. FEBS J..

[27] Li Y., Jiao J. (2020). Deficiency of TRPM2 leads to embryonic neurogenesis defects in hyperthermia. Sci. Adv..

[28] Ngoc Tran T.D., Stovall K.E., Suantawee T., Hu Y., Yao S., Yang L.J., Adisakwattana S., Cheng H. (2017). Transient receptor potential melastatin 4 channel is required for rat dental pulp stem cell proliferation and survival. Cell Prolif..

[29] Cui L., Xu S.M., Ma D.D., Wu B.L. (2014). The effect of TRPM7 suppression on the proliferation, migration and osteogenic differentiation of human dental pulp stem cells. Int. Endod. J..

[30] De Clercq K., Perez-Garcia V., Van Bree R., Pollastro F., Peeraer K., Voets T., Vriens J. (2021). Mapping the expression of transient receptor potential channels across murine placental development. Cell. Mol. Life Sci..

[31] Zou Z.G., Rios F.J., Montezano A.C., Touyz R.M. (2019). TRPM7, Magnesium, and Signaling. Int. J. Mol. Sci..

[32] Hong F., Wu S., Zhang C., Li L., Chen J., Fu Y., Wang J. (2020). TRPM7 Upregulate the Activity of SMAD1 through PLC Signaling Way to Promote Osteogenesis of hBMSCs. BioMed Res. Int..

[33] Cheng H., Feng J.M., Figueiredo M.L., Zhang H., Nelson P.L., Marigo V., Beck A. (2010). Transient receptor potential melastatin type 7 channel is critical for the survival of bone marrow derived mesenchymal stem cells. Stem Cells Dev..

[34] Castiglioni S., Romeo V., Locatelli L., Zocchi M., Zecchini S., Maier J.A.M. (2019). The simultaneous downregulation of TRPM7 and MagT1 in human mesenchymal stem cells in vitro: Effects on growth and osteogenic differentiation. Biochem. Biophys. Res. Commun..

[35] Xiao E., Chen C., Zhang Y. (2016). The mechanosensor of mesenchymal stem cells: Mechanosensitive channel or cytoskeleton?. Stem Cell Res. Ther..

[36] Xiao E., Yang H.Q., Gan Y.H., Duan D.H., He L.H., Guo Y., Wang S.Q., Zhang Y. (2015). Brief reports: TRPM7 Senses mechanical stimulation inducing osteogenesis in human bone marrow mesenchymal stem cells. Stem Cells.

[37] Swain S.M., Parameswaran S., Sahu G., Verma R.S., Bera A.K. (2012). Proton-gated ion channels in mouse bone marrow stromal cells. Stem Cell Res..

[38] Bevan S., Quallo T., Andersson D.A. (2014). Trpv1. Mammalian Transient Receptor Potential (TRP) Cation Channels, Handbook of Experimental Pharmacology.

[39] Edwards J.G. (2014). TRPV1 in the central nervous system: Synaptic plasticity, function, and pharmacological implications. Prog. Drug Res..

[40] Ramirez-Barrantes R., Cordova C., Poblete H., Munoz P., Marchant I., Wianny F., Olivero P. (2016). Perspectives of TRPV1 Function on the Neurogenesis and Neural Plasticity. Neural Plast..

[41] Santoni G., Nabissi M., Amantini C., Santoni M., Ricci-Vitiani L., Pallini R., Maggi F., Morelli M.B. (2021). ERK Phosphorylation Regulates the Aml1/Runx1 Splice Variants and the TRP Channels Expression during the Differentiation of Glioma Stem Cell Lines. Cells.

[42] Sviderskaya E.V., Easty D.J., Lawrence M.A., Sanchez D.P., Negulyaev Y.A., Patel R.H., Anand P., Korchev Y.E., Bennett D.C. (2009). Functional neurons and melanocytes induced from immortal lines of postnatal neural crest-like stem cells. FASEB J..

[43] Stein A.T., Ufret-Vincenty C.A., Hua L., Santana L.F., Gordon S.E. (2006). Phosphoinositide 3-kinase binds to TRPV1 and mediates NGF-stimulated TRPV1 trafficking to the plasma membrane. J. Gen. Physiol..

[44] Zeng Y., Han H., Tang B., Chen J., Mao D., Xiong M. (2018). Transplantation of Recombinant Vascular Endothelial Growth Factor (VEGF)189-Neural Stem Cells Downregulates Transient Receptor Potential Vanilloid 1 (TRPV1) and Improves Motor Outcome in Spinal Cord Injury. Med. Sci. Monit..

[45] Arimura Y., Shindo Y., Yamanaka R., Mochizuki M., Hotta K., Nakahara T., Ito E., Yoshioka T., Oka K. (2021). Peripheral-neuron-like properties of differentiated human dental pulp stem cells (hDPSCs). PLoS ONE.

[46] Kurosaka M., Ogura Y., Funabashi T., Akema T. (2016). Involvement of Transient Receptor Potential Cation Channel Vanilloid 1 (TRPV1) in Myoblast Fusion. J. Cell. Physiol..

[47] Wang Y., Huang Y.Y., Wang Y., Lyu P., Hamblin M.R. (2017). Red (660 nm) or near-infrared (810 nm) photobiomodulation stimulates, while blue (415 nm), green (540 nm) light inhibits proliferation in human adipose-derived stem cells. Sci. Rep..

[48] Voets T., Prenen J., Vriens J., Watanabe H., Janssens A., Wissenbach U., Bodding M., Droogmans G., Nilius B. (2002). Molecular determinants of permeation through the cation channel TRPV4. J. Biol. Chem..

[49] Everaerts W., Nilius B., Owsianik G. (2010). The vanilloid transient receptor potential channel TRPV4: From structure to disease. Prog. Biophys. Mol. Biol..

[50] Corrigan M.A., Johnson G.P., Stavenschi E., Riffault M., Labour M.N., Hoey D.A. (2018). TRPV4-mediates oscillatory fluid shear mechanotransduction in mesenchymal stem cells in part via the primary cilium. Sci. Rep..

[51] Gilchrist C.L., Leddy H.A., Kaye L., Case N.D., Rothenberg K.E., Little D., Liedtke W., Hoffman B.D., Guilak F. (2019). TRPV4-mediated calcium signaling in mesenchymal stem cells regulates aligned collagen matrix formation and vinculin tension. Proc. Natl. Acad. Sci. USA.

[52] Das R., Goswami C. (2019). TRPV4 expresses in bone cell lineages and TRPV4-R616Q mutant causing Brachyolmia in human reveals “loss-of-interaction” with cholesterol. Biochem. Biophys. Res. Commun..

[53] Jin S.S., He D.Q., Wang Y., Zhang T., Yu H.J., Li Z.X., Zhu L.S., Zhou Y.H., Liu Y. (2020). Mechanical force modulates periodontal ligament stem cell characteristics during bone remodelling via TRPV4. Cell Prolif..

[54] Xu C., Luo J., He L., Montell C., Perrimon N. (2017). Oxidative stress induces stem cell proliferation via TRPA1/RyR-mediated Ca(^2+^) signaling in the Drosophila midgut. eLife.

[55] Chen C., Huang X., Zhu W., Ding C., Huang P., Li R. (2021). H_2_O_2_ gel bleaching induces cytotoxicity and pain conduction in dental pulp stem cells via intracellular reactive oxygen species on enamel/dentin disc. PLoS ONE.

[56] Terrie E., Deliot N., Benzidane Y., Harnois T., Cousin L., Bois P., Oliver L., Arnault P., Vallette F., Constantin B. (2021). Store-Operated Calcium Channels Control Proliferation and Self-Renewal of Cancer Stem Cells from Glioblastoma. Cancers.

[57] Liu M., Inoue K., Leng T., Guo S., Xiong Z.G. (2014). TRPM7 channels regulate glioma stem cell through STAT3 and Notch signaling pathways. Cell. Signal..

[58] Liu K., Xu S.H., Chen Z., Zeng Q.X., Li Z.J., Chen Z.M. (2018). TRPM7 overexpression enhances the cancer stem cell-like and metastatic phenotypes of lung cancer through modulation of the Hsp90alpha/uPA/MMP2 signaling pathway. BMC Cancer.

[59] Nabissi M., Morelli M.B., Arcella A., Cardinali C., Santoni M., Bernardini G., Santoni A., Santoni G., Amantini C. (2016). Post-transcriptional regulation of 5′-untranslated regions of human Transient Receptor Potential Vanilloid type-1 (TRPV-1) channels: Role in the survival of glioma patients. Oncotarget.

[60] Nabissi M., Morelli M.B., Amantini C., Liberati S., Santoni M., Ricci-Vitiani L., Pallini R., Santoni G. (2015). Cannabidiol stimulates Aml-1a-dependent glial differentiation and inhibits glioma stem-like cells proliferation by inducing autophagy in a TRPV2-dependent manner. Int. J. Cancer.

[61] Morelli M.B., Nabissi M., Amantini C., Farfariello V., Ricci-Vitiani L., di Martino S., Pallini R., Larocca L.M., Caprodossi S., Santoni M. (2012). The transient receptor potential vanilloid-2 cation channel impairs glioblastoma stem-like cell proliferation and promotes differentiation. Int. J. Cancer.

[62] Hu Z., Cao X., Fang Y., Liu G., Xie C., Qian K., Lei X., Cao Z., Du H., Cheng X. (2018). Transient receptor potential vanilloid-type 2 targeting on stemness in liver cancer. Biomed. Pharmacother..

[63] Guo Q., Xiao X.Y., Wu C.Y., Li D., Chen J.L., Ding X.C., Cheng C., Chen C.R., Tong S., Wang S.H. (2022). Clinical Roles of Risk Model Based on Differentially Expressed Genes in Mesenchymal Stem Cells in Prognosis and Immunity of Non-Small Cell Lung Cancer. Front. Genet..

[64] Shin H.Y., Hong Y.H., Jang S.S., Chae H.G., Paek S.L., Moon H.E., Kim D.G., Kim J., Paek S.H., Kim S.J. (2010). A role of canonical transient receptor potential 5 channel in neuronal differentiation from A2B5 neural progenitor cells. PLoS ONE.

[65] Louhivuori L.M., Jansson L., Turunen P.M., Jantti M.H., Nordstrom T., Louhivuori V., Akerman K.E. (2015). Transient receptor potential channels and their role in modulating radial glial-neuronal interaction: A signaling pathway involving mGluR5. Stem Cells Dev..

[66] Li M., Chen C., Zhou Z., Xu S., Yu Z. (2012). A TRPC1-mediated increase in store-operated Ca^2+^ entry is required for the proliferation of adult hippocampal neural progenitor cells. Cell Calcium.

[67] Stanslowsky N., Tharmarasa S., Staege S., Kalmbach N., Klietz M., Schwarz S.C., Leffler A., Wegner F. (2018). Calcium, Sodium, and Transient Receptor Potential Channel Expression in Human Fetal Midbrain-Derived Neural Progenitor Cells. Stem Cells Dev..

[68] Kuang C.Y., Yu Y., Wang K., Qian D.H., Den M.Y., Huang L. (2012). Knockdown of transient receptor potential canonical-1 reduces the proliferation and migration of endothelial progenitor cells. Stem Cells Dev..

[69] Du L.L., Shen Z., Li Z., Ye X., Wu M., Hong L., Zhao Y. (2018). TRPC1 Deficiency Impairs the Endothelial Progenitor Cell Function via Inhibition of Calmodulin/eNOS Pathway. J. Cardiovasc. Transl. Res..

[70] Ong E.C., Nesin V., Long C.L., Bai C.X., Guz J.L., Ivanov I.P., Abramowitz J., Birnbaumer L., Humphrey M.B., Tsiokas L. (2013). A TRPC1 protein-dependent pathway regulates osteoclast formation and function. J. Biol. Chem..

[71] Morgan P.J., Hubner R., Rolfs A., Frech M.J. (2013). Spontaneous calcium transients in human neural progenitor cells mediated by transient receptor potential channels. Stem Cells Dev..

[72] Stock K., Kumar J., Synowitz M., Petrosino S., Imperatore R., Smith E.S., Wend P., Purfurst B., Nuber U.A., Gurok U. (2012). Neural precursor cells induce cell death of high-grade astrocytomas through stimulation of TRPV1. Nat. Med..

[73] Dragoni S., Guerra G., Fiorio Pla A., Bertoni G., Rappa A., Poletto V., Bottino C., Aronica A., Lodola F., Cinelli M.P. (2015). A functional transient receptor potential vanilloid 4 (TRPV4) channel is expressed in human endothelial progenitor cells. J. Cell. Physiol..

[74] Chen C., Ma Q., Deng P., Yang J., Yang L., Lin M., Yu Z., Zhou Z. (2017). Critical role of TRPC1 in thyroid hormone-dependent dopaminergic neuron development. Biochim. Biophys. Acta Mol. Cell Res..

[75] Hao H.B., Webb S.E., Yue J., Moreau M., Leclerc C., Miller A.L. (2018). TRPC3 is required for the survival, pluripotency and neural differentiation of mouse embryonic stem cells (mESCs). Sci. China Life Sci..

[76] Eapen A., Kulkarni R., Ravindran S., Ramachandran A., Sundivakkam P., Tiruppathi C., George A. (2013). Dentin phosphophoryn activates Smad protein signaling through Ca^2+^-calmodulin-dependent protein kinase II in undifferentiated mesenchymal cells. J. Biol. Chem..

[77] Nelson P., Ngoc Tran T.D., Zhang H., Zolochevska O., Figueiredo M., Feng J.M., Gutierrez D.L., Xiao R., Yao S., Penn A. (2013). Transient receptor potential melastatin 4 channel controls calcium signals and dental follicle stem cell differentiation. Stem Cells.

[78] Wan J., Guo A.A., King P., Guo S., Saafir T., Jiang Y., Liu M. (2020). TRPM7 Induces Tumorigenesis and Stemness through Notch Activation in Glioma. Front. Pharmacol..

[79] Middelbeek J., Visser D., Henneman L., Kamermans A., Kuipers A.J., Hoogerbrugge P.M., Jalink K., van Leeuwen F.N. (2015). TRPM7 maintains progenitor-like features of neuroblastoma cells: Implications for metastasis formation. Oncotarget.

[80] Cobaleda C., Perez-Caro M., Vicente-Duenas C., Sanchez-Garcia I. (2007). Function of the zinc-finger transcription factor SNAI2 in cancer and development. Annu. Rev. Genet..

[81] Ying Z., Li Y., Wu J., Zhu X., Yang Y., Tian H., Li W., Hu B., Cheng S.Y., Li M. (2013). Loss of miR-204 expression enhances glioma migration and stem cell-like phenotype. Cancer Res..

[82] Courboulin A., Paulin R., Giguere N.J., Saksouk N., Perreault T., Meloche J., Paquet E.R., Biardel S., Provencher S., Cote J. (2011). Role for miR-204 in human pulmonary arterial hypertension. J. Exp. Med..

[83] Kim H.Y., Yu Y., Oh S.Y., Wang K.K., Kim Y.R., Jung S.C., Kim H.S., Jo I. (2019). Far-Infrared Irradiation Inhibits Adipogenic Differentiation and Stimulates Osteogenic Differentiation of Human Tonsil-Derived Mesenchymal Stem Cells: Role of Protein Phosphatase 2B. Cell Physiol. Biochem..

[84] Kim H.Y., Oh S.Y., Choi Y.M., Park J.H., Kim H.S., Jo I. (2021). Transient receptor potential vanilloid 2 mediates the inhibitory effect of far-infrared irradiation on adipogenic differentiation of tonsil-derived mesenchymal stem cells. Stem Cell Res..

[85] Hu K., Sun H., Gui B., Sui C. (2017). TRPV4 functions in flow shear stress induced early osteogenic differentiation of human bone marrow mesenchymal stem cells. Biomed. Pharmacother..

[86] Winslow M.M., Pan M., Starbuck M., Gallo E.M., Deng L., Karsenty G., Crabtree G.R. (2006). Calcineurin/NFAT signaling in osteoblasts regulates bone mass. Dev. Cell.

[87] Masuyama R., Vriens J., Voets T., Karashima Y., Owsianik G., Vennekens R., Lieben L., Torrekens S., Moermans K., Vanden Bosch A. (2008). TRPV4-mediated calcium influx regulates terminal differentiation of osteoclasts. Cell Metab..

[88] He L.H., Liu M., He Y., Xiao E., Zhao L., Zhang T., Yang H.Q., Zhang Y. (2017). TRPV1 deletion impaired fracture healing and inhibited osteoclast and osteoblast differentiation. Sci. Rep..

[89] Fiorio Pla A., Ong H.L., Cheng K.T., Brossa A., Bussolati B., Lockwich T., Paria B., Munaron L., Ambudkar I.S. (2012). TRPV4 mediates tumor-derived endothelial cell migration via arachidonic acid-activated actin remodeling. Oncogene.

[90] Thodeti C.K., Matthews B., Ravi A., Mammoto A., Ghosh K., Bracha A.L., Ingber D.E. (2009). TRPV4 channels mediate cyclic strain-induced endothelial cell reorientation through integrin-to-integrin signaling. Circ. Res..

[91] Amcheslavsky A., Jiang J., Ip Y.T. (2009). Tissue damage-induced intestinal stem cell division in Drosophila. Cell Stem Cell.

[92] Ohlstein B., Spradling A. (2006). The adult Drosophila posterior midgut is maintained by pluripotent stem cells. Nature.

[93] Qian F., Germino F.J., Cai Y., Zhang X., Somlo S., Germino G.G. (1997). PKD1 interacts with PKD2 through a probable coiled-coil domain. Nat. Genet..

[94] Jung S.H., You J.E., Choi S.W., Kang K.S., Cho J.Y., Lyu J., Kim P.H. (2021). Polycystin-1 Enhances Stemmness Potential of Umbilical Cord Blood-Derived Mesenchymal Stem Cells. Int. J. Mol. Sci..

[95] Cheng W., Zheng J. (2021). Distribution and Assembly of TRP Ion Channels. Adv. Exp. Med. Biol..

